# Risk factors associated with severe adverse events in patients with relapsing polychondritis undergoing flexible bronchoscopy

**DOI:** 10.1186/s13023-024-03061-9

**Published:** 2024-02-09

**Authors:** Shao-Ting Wang, Jinglan Wang, Xiaoxing Gao, Keqi Chen, Kai-Feng Xu, Xinlun Tian

**Affiliations:** 1 Department of Pulmonary and Critical Care Medicine, Peking Union Medical College Hospital, Chinese Academy of Medical Sciences, #1 Shuaifuyuan Hutong, Beijing, 100730 China; 2 State Key Laboratory of Complex Severe and Rare Diseases, Peking Union Medical College Hospital, Beijing, 100730 China

**Keywords:** Relapsing polychondritis, Respiratory involvement, Spirometry, Bronchoscopy, Severe adverse event

## Abstract

**Background:**

Patients with relapsing polychondritis (RP) sometimes experience upper airway collapse or lower airway stenosis, and bronchoscopy may provide a valuable typical image to confirm the diagnosis. This study aimed to identify potential risk factors associated with severe adverse effects during bronchoscopy.

**Methods:**

We performed a retrospective cohort study of 82 consecutive patients with RP hospitalized at Peking Union Medical College Hospital between January 1, 2012 and December 31, 2022. Clinical features and disease patterns were compared among patients with RP undergoing bronchoscopy with or without severe adverse effects. Binary logistic regression analysis was performed to identify the associated risk factors.

**Results:**

For patients with RP undergoing bronchoscopy with severe adverse effects, the forced vital capacity (FVC), forced vital capacity percent predicted values (FVC%), and peak expiratory flow were significantly lower (*P* = 0.001, *P* = 0.001, and *P* = 0.021, respectively) than those in the non-severe adverse effect subgroup. Binary logistic regression analysis revealed that low FVC% (odds ratio, 0.930; 95% confidence interval, 0.880–0.982; *P* = 0.009) was an independent risk factor for severe adverse events in patients undergoing bronchoscopy.

**Conclusions:**

Low FVC or FVC% suggests a high risk of severe adverse effects in patients with RP undergoing bronchoscopy. Patients with such risk factors should be carefully evaluated before bronchoscopy and adequately prepared for emergency tracheal intubation or tracheostomy.

**Supplementary Information:**

The online version contains supplementary material available at 10.1186/s13023-024-03061-9.

## Introduction

Relapsing polychondritis (RP) is a rare systemic autoimmune disease characterized by recurrent inflammation and progressive destruction of cartilaginous tissues, predominantly in the external ear, nose, and tracheobronchial tree. Studies have reported that 21–50% of patients have airway involvement, including upper airway collapse and stenosis of the lower airway cartilage, and tracheobronchomalacia (TBM) is the most serious complication [1]. Tracheobronchial lesions may cause recurrent, potentially severe, lower respiratory tract infections and fatal obstructive respiratory failure.

Patients with RP may experience dyspnea, persistent cough, and shortness of breath, which are often misdiagnosed as asthma or chronic bronchitis with inappropriate therapy, and misdiagnosis and long-term diagnosis delay may lead to poor prognosis. Bronchoscopy is important for the diagnosis of RP because it can evaluate mucositis, define the severity of airway stenosis, and allow the dynamic assessment of possible collapse, as the gold standard criterion of TBM is the reduction of the cross-sectional area of the trachea or bronchus lumen by at least 50% at the end of expiration or coughing compared with the inspiratory phase during bronchoscopy. However, bronchoscopy in patients with RP can be fatally risky, leading to acute edema of the respiratory or supraglottic airways, bronchospasm, respiratory distress, or cardiac insufficiency, as conventional mechanical ventilation and general anesthesia often cannot guarantee oxygen supply or safety.

In this study, we retrospectively compared patients with RP who underwent bronchoscopy with or without severe adverse effects to identify the potential risk factors associated with severe adverse effects.

## Methods

### Study design and patients

We conducted a retrospective, observational study at Peking Union Medical College Hospital (PUMCH) for clinical assessment in which all 1,407 patients were consecutively diagnosed with RP according to the diagnostic criteria of the McAdam criteria [2] between January 1, 2012 and December 31, 2022, while 395 (28.1%) of patients had trachea or bronchus involvement. Eighty-two patients who underwent bronchoscopy during this period were included. The indications for bronchoscopy included (1) to obtain samples of etiologic detection; (2) to assess typical microscopic changes of RP and define the severity of airway stenosis, (3) or to rule out alternative diagnoses such as amyloidosis by mucosal biopsy. The study protocol was approved by the ethical committee of PUMCH (I22-PJ1043) and performed in accordance with the Declaration of Helsinki. Written consent was obtained from all patients.

### Data collection

Baseline characteristics of the patients were collected upon admission, including demographics, clinical features, comorbidities, spirometry, and inflammatory markers, including erythrocyte sedimentation rate (ESR) and high-sensitivity C-reactive protein (hsCRP). Forced Expiratory volume in 1 s (FEV_1_), forced expiratory volume in 1 s percent predicted values (FEV_1_%), forced vital capacity (FVC), forced vital capacity percent predicted values (FVC%), forced expiratory volume in 1 s to forced vital capacity ratio (FEV_1_/FVC), peak expiratory flow (PEF), and peak expiratory flow percent predicted values (PEF%) of spirometry were collected within two weeks before bronchoscopy. Furthermore, flexible bronchoscopy was performed in all patients to diagnose RP or evaluate the lesions. Flexible bronchoscopy (BF-240 bronchoscope, Olympus, Japan) was performed in all patients to examine the tracheobronchial tree. All patients received local anesthesia with 5mL 2% lidocaine solution via nebulizer for 30 min before bronchoscopy. In supine position without head elevation, bronchoscopy was performed trans-nasally or trans-orally and 2% lidocaine solution was administrated for local anesthesia of the vocal cord and bronchial tree. We analyzed the severe adverse events after bronchoscopy. The severe adverse events were defined as (1) bronchospasm or laryngospasm, which needs urgent use of salbutamol, epinephrine, or succinylcholine to treat acute respiratory events; (2) arrhythmia or hypotension during bronchoscopy; (3) upgraded respiratory support caused by severe hypoxemia, which includes the need for additional supplemental oxygen (such as a nasal cannula or high flow), positive airway pressure therapy, or unplanned inability to extubate at the end of the procedure, reintubated within 24 h of procedure, or higher ventilator settings than baseline not previously prescribed; (4) aspiration pneumonia; (5) newly onset of pneumothorax or mediastinal emphysema confirmed by thoracic imaging; and (6) unplanned emergency admissions. The primary endpoint was to assess the predictive value of the risk factors associated with severe adverse events in patients with RP undergoing flexible bronchoscopy.

### Statistical analysis

Statistical analyses were performed using statistical software programs (PASW statistics 22.0; SPSS Inc., Chicago, IL., USA). Means and percentages are presented as appropriate. Differences in demographic and clinical characteristics between patients with or without severe adverse events of bronchoscopy were summarized using the Student’s t-test or Wilcoxon rank-sum test for continuous variables and the Pearson χ^2^test or Fisher’s exact test for categorical variables. Variables with *p*-values less than 0.1 were entered into the initial binary model. Binary logistic regression analysis was performed to identify the factors associated with severe adverse events during flexible bronchoscopy. A statistical *p*-value less than 0.05 was considered statistically significant.

## Results

A total of 82 patients with RP were identified, 17 (20.7%) of whom developed severe adverse events as we defined during flexible bronchoscopy. 18 patients received bronchoalveolar lavage, 16 received endobronchial biopsy in group without severe adverse events. 3 patients received bronchoalveolar lavage, 2 received endobronchial biopsy in group with severe adverse events. We found no relation with the severe adverse events and such operations during the bronchoscopy. The most common comorbidity of the patients was acute respiratory infection, mentioned in Table 1. Other comorbidity included one Chronic Hepatitis B, one Sjögren’s syndrome, one psoriasis, autoimmune encephalitis in group without severe adverse events, and one common variable immunodeficiency, one psoriasis, two chronic thyroiditis in group with severe adverse events. After bronchoscopy, we found the location of the lesion is only bronchus in twenty-three patients, while both trachea and bronchus in thirty-eight patients in group without severe adverse events; both trachea and bronchus in sixteen patients in group with severe adverse events. Demographic data, exposure variables, and univariate logistic regression analyses of probable risk factors are shown in Table 1. To compare patients with and without severe adverse events, low FVC, FVC%, and PEF were identified as risk factors for severe adverse events during bronchoscopy. Female sex, advanced age, low body mass index (BMI), and PEF% were associated with severe adverse events, but there was no statistical significance. The details of severe adverse events for each of 17 patients were listed in Table 2.

**Table 1:** Demographics and clinical characteristics of patients undergoing bronchoscopy

Variable	Bronchoscopy with severe adverse events (*n* = 17)	Bronchoscopy without severe adverse events (*n* = 65)	Statistic	p-value
Male sex	6 (35.3%)	38 (58.5%)	χ^2^ = 2.909	0.088
Age (years)	51.2 ± 10.1	46.0 ± 12.7	t = 1.766	0.081
BMI (kg/m^2^)	20.6 [17.6–24.1]	22.3 [20.4–26.3]	Z=-1.838	0.066
Acute respiratory infection^#^	4 (23.5%)	13 (20%)	χ^2^ = 0.102	0.749
Current use of Glucocorticoid	9 (52.9%)	32 (49.2%)	χ^2^ = 0.074	0.785
ESR (mm/h)	66 ± 44.2	52.5 [15.3–91]	Z=-0.910	0.363
hsCRP (mg/L)	33.9 [9.3–110.5]	21.1 [2–85.5]	Z=-0.938	0.348
pO_2_ (mmHg)	85.8 ± 19.1	87.1 ± 15.4	t = 0.219	0.828
pCO_2_ (mmHg)	39.9 ± 5.1	39.3 ± 3.6	t=-0.459	0.649
FEV_1_ (L)	1.2 [0.5–1.6]	1.4 ± 0.7	Z=-0.912	0.362
FEV_1_%	37.4 ± 22.0	47.7 ± 19.5	t = 1.624	0.109
FVC (L)	2.3 ± 0.7	3.1 ± 0.7	t = 3.553	0.001^*^
FVC%	69.4 ± 18.2	87.7 ± 16.5	t = 3.417	0.001^*^
FEV_1_/FVC	47.6 ± 16.1	47.6 [34.2–60.4]	Z=-0.467	0.640
PEF(L/s)	1.5 [1.3–2.3]	2.6 [1.8–4.1]	Z=-2.314	0.021^*^
PEF%	22.8 [16.6–39.5]	36.8 [24.6–53.0]	Z=-1.872	0.061
Bronchoalveolar lavage	3(17.6%)	18(27.7%)	χ^2^ = 0.284	0.594
Endobronchial biopsy	2(11.7%)	16(24.6%)	χ^2^ = 0.657	0.418

Data are presented as the mean + standard deviation, number (%), or median [interquartile range]

^#^ Acute infection confirmed by recent CT tomography or lower tract respiratory specimens

^*^p < 0.05

**Table 2:** Details of severe adverse events in the 17 patients undergoing bronchoscopy

Patient	bronchospasm or laryngospasm	arrhythmia or hypotension	upgraded respiratory support	aspiration pneumonia	pneumothorax or mediastinal emphysema	unplanned emergency admissions
1	√					
2	√		√	√		
3	√					
4	√					
5	√					
6	√	√				√
7	√					
8	√					
9	√					
10	√					
11	√					
12	√					
13	√					
14	√					
15	√					
16	√	√	√			
17		√				

We enrolled the parameter of *p*-value less than 0.10 from the Table 1 to perform the binary logistic regression analysis. The results are shown in Table 3. Since multicollinearity was detected between FVC and FVC%, FVC was excluded from the binary variable analysis. PEF was removed from the analysis because of multicollinearity, which indicated that only low FVC% (odds ratio [OR], 0.930; 95% confidence interval [CI], 0.880–0.982; *p* = 0.009) was an independent risk factor for severe adverse events in patients undergoing bronchoscopy. A *p*-value of the Hosmer–Lemeshow test was 0.512.

**Table 3:** Binary logistic regression analysis of variables predicting severe adverse events undergoing bronchoscopy

Variable	Adjusted OR (95% CI)	p-value
Male sex	0.088 (0.006–1.221)	0.070
Age	1.066 (0.942–1.206)	0.308
BMI	0.785 (0.584–1.054)	0.107
FVC%	0.930 (0.880–0.982)	0.009^*^
PEF%	0.921 (0.811–1.045)	0.200

^*^p < 0.05

**Fig. 1:**
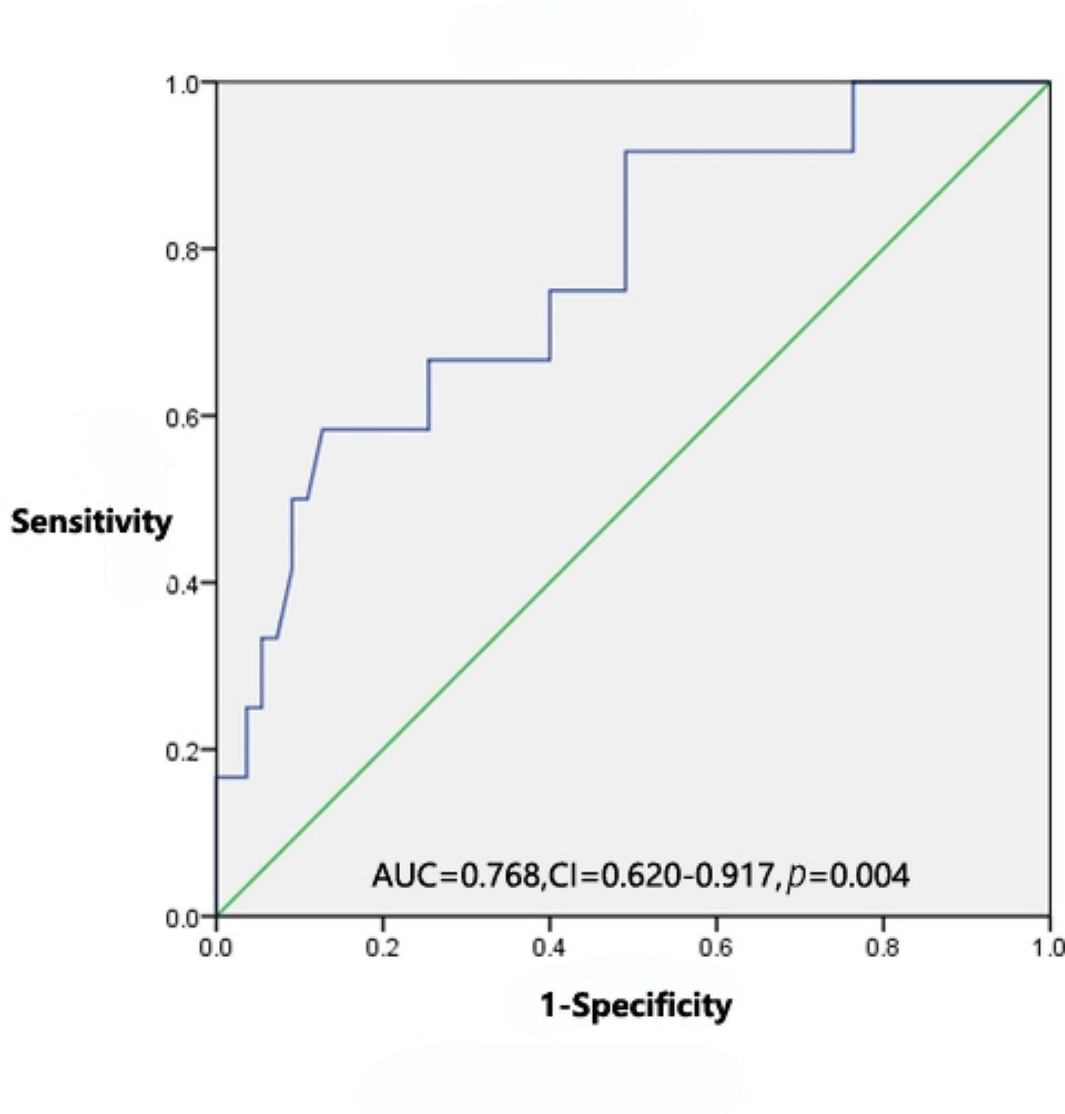
ROC curve of FVC% and severe adverse events to assess the optimal cut-off values of FVC%

Figure 1 shows the receiver operating characteristic (ROC) curves for FVC% and severe adverse events. When the FVC% was 51.93%, the probability of severe adverse events was approximately 50%. While calculating the Yoden index with a maximum of 0.456, the cut-off value of FVC% for predicting severe adverse events was 70.65%, and the sensitivity and specificity for severe adverse events were 87.3% and 58.3%, respectively (area under the ROC curve, 0.768; 95% CI, 0.620–0.917; *p* = 0.004).

## Discussion

This study aimed to predict the possibility of severe complications of bronchoscopy in patients using FVC measurements. We found that patients with RP who had lower FVC or FVC% had a high risk of severe adverse events during bronchoscopy.

Dion identified three RP phenotypes: hematological, respiratory, and mild, and discovered that 22% of patients with RP exhibited respiratory manifestations, and this subgroup suffered more infections and respiratory insufficiency with high mortality [3, 4]. Patients with RP undergoing bronchoscopy could be at risk, but there is a lack of studies investigating the incidence or risk factors of severe adverse events in patients undergoing bronchoscopy. Only one study has reported the case of a 35-year-old male patient with RP who was scheduled for rigid bronchoscopy to relieve dyspnea caused by subglottic stenosis. After laser splitting of the subglottic web, the patient’s spontaneous respiration was insufficient, and hypercarbia progressively developed even with assisted ventilation [5].

Female patients, advanced age, and low BMI were prone to have a high incidence of severe adverse events during bronchoscopy, suggesting that female patients with low BMI may suffer from high airway hyperresponsiveness and are more susceptible to bronchospasm and laryngospasm, whereas advanced age indicates a long clinical course or potential cardiorespiratory instability. Bronchoscopy with such risk factors should be performed with care and only by an experienced interventional endoscopist with trained personnel and a nearby emergency airway and tracheostomy kit.

In addition, to avoid severe adverse events after bronchoscopy, patients with RP should undergo spirometry and volume-flow curves before bronchoscopy. Pulmonary function tests can provide valuable information for patients with RP about the presence of fixed airway stenosis or dynamic airway closure, or it can illustrate the severity of airway limitation. The process of RP airway involvement is considered to be inflammatory edema, collapse related to malacia, and fibrous stenosis. There are three presentations of obstruction: fixed obstruction with a double plateau volume-flow curve (expiratory and inspiratory involvement), variable or extrathoracic dynamic obstruction corresponding to the upper airways (extrathoracic trachea or larynx or inspiratory involvement), and variable or dynamic intrathoracic obstruction (expiratory involvement) [6, 7]. The flow-volume curve of TBM is characterized by a decrease in the flow rate from the peak flow to an inflection point with a peak flow rate < 50%, which occurs within the first 25% of the expired vital capacity, indicating that bronchoscopy is usually dangerous [8]. We also found that a low FVC, FVC%, or PEF was associated with respiratory distress during bronchoscopy and that a reduction in FVC or PEF was mainly due to severe obstructive disease, while we did not find that decreased total lung capacity (TLC) and FEV_1_ have any relevance to severe adverse events.

To be airway disease involving mainly small airways, meta-analysis of diagnostic bronchoscopy in patients with COPD showed the overall major complication rate of bronchoscopy was 4.3% (including hemoptysis requiring observation or intervention, pneumothorax, hemodynamic instability requiring vasoactive drugs, respiratory failure, or asphyxia, et al., 95% CI, 2.2-8.2%; 18 trials/arms, *n* = 2000). The major complication rate of the patients with an exacerbation of chronic obstructive pulmonary disease (COPD) was higher than that of the stable patients (7.8% vs. 4.5%, *p* < 0.01). While using sedative medicine (*P* = 0.043), patients who were GOLD stages III and IV [9] (*p* < 0.01) or had a high BMI (*p* < 0.01) more easily encountered complications [10]. We compared RP with COPD patients who underwent bronchoscopy, while they may be not comparable, as the most common severe adverse events of RP were bronchospasm or laryngospasm, while the major complication of COPD patients who underwent bronchoscopy recorded were respiratory failure and hemoptysis. We divided our RP cohort patients using the GOLD grading of COPD and classified our 82 patients into 4 subgroups. The proportion of severe adverse events of our patients with GOLD 1, 2, 3, 4 divided by FEV1% were 20.0% (1/5), 13.0% (3/23), 14.8% (4/27) and 26.6% (4/15), respectively, which was much higher compared with recent published data in COPD. The proportion of severe adverse events in COPD who underwent bronchoscopy was 3% (1 in 35 GOLD1 or GOLD2), and 9% (3 in 32 GOLD3 or GOLD4) from previous study [10]. For asthma, adverse events occurred on 34 of 273 occasions (12.5%), while bronchospasm or worsening of asthma symptoms occurred on 14 occasions (7.3%) [11, 12]. Spirometry assessment revealed that the decrease in FEV_1_ following bronchoscopy was similar in severe and milder asthma groups [13]. However, for RP, the larynx and tracheobronchial tree manifestations always involve the large airways, and FVC may be a better index to evaluate the large airways than FEV_1_.

In addition, to prevent patients with RP with extremely low FVC% from undergoing bronchoscopy and preparing for emergency tracheal intubation or tracheostomy, there are other preventive measures. Studies have reported that dyspnea can be aggravated when lying in a supine position, presumably due to airway collapse, which may help relieve respiratory symptoms, and spirometry and oximetry performed both upright and supine may be useful [14].

Our study had some limitations. This was a single-center, retrospective study with a small sample size, and more multicenter studies are needed to validate the conclusions of this study.

## Conclusions

We found that a low FVC% was a risk factor for patients with RP undergoing bronchoscopy with severe adverse events. Patients with RP should be carefully evaluated, especially with spirometry measurements before bronchoscopy, to weigh the pros and cons, with adequate preparation for emergency tracheal intubation or tracheostomy.

### Electronic supplementary material

Below is the link to the electronic supplementary material.

Supplementary Material 1

## Data Availability

The authors confirm that the data supporting the findings of this study are available within the article.

## Abbreviations

BMI: Body mass index
CI: Confidence interval
ESR: Erythrocyte sedimentation rate
FEV_1_: Forced expiratory volume in 1s
FEV_1_%: Forced expiratory volume in 1 s percent predicted values
FVC: Forced vital capacity
FVC%: Forced expiratory volume percent predicted values
FEV_1_/FVC: Forced expiratory volume in 1s to forced vital capacity ratio
hsCRP: High-sensitivity C-reactive protein
IQR: Interquartile range
OR: Odds ratio
pCO_2_: Partial pressure of carbon dioxide
PEF: Peak expiratory flow
PEF%: Peak expiratory flow percent predicted values
pO_2_: Partial pressure of oxygen
ROC: Receiver operating characteristic
RP: Relapsing polychondritis
SD: Standard deviation
TBM: Tracheobronchomalacia
TLC: Total lung capacity
