# Supplementary material for: Risk factors associated with severe adverse events in patients with relapsing polychondritis undergoing flexible bronchoscopy
Source: Orphanet J Rare Dis. 2024 Feb 9;19:54. doi: 10.1186/s13023-024-03061-9 (PMC10858597; doi:10.1186/s13023-024-03061-9)
Supplement: Supplementary file 1 — Supplementary Material 1 [file 13023_2024_3061_MOESM1_ESM.pdf]

# Certificate of Elsevier Language Editing Services

## The following article was edited by Elsevier Language Editing Services:

**Risk factors associated with severe adverse events in relapsing polychondritis patients undergoing flexible bronchoscopy**

## Ordered by:

**Shao-Ting Wang**

### Estimated Delivery date:

**2023-06-20**

### Order reference:

**ASLESTD0508700**
